# Potential Therapeutic Efficacy of Ferulic Acid and Its Derivatives in the Management of Cancers: A Comprehensive Analysis With Mechanistic Insight

**DOI:** 10.1155/ijfo/2256871

**Published:** 2025-05-30

**Authors:** Shakil Ahmmed, Md. Shimul Bhuia, Raihan Chowdhury, Md. Hanif Munshi, Tanzila Akter Eity, Hossam Kamli, Muhammad Torequl Islam

**Affiliations:** ^1^Department of Biochemistry and Molecular Biology, Bangladesh Agricultural University, Mymensingh, Bangladesh; ^2^Department of Pharmacy, Gopalganj Science and Technology University, Gopalganj, Bangladesh; ^3^Department of Textile Engineering, Uttara University, Dhaka, Bangladesh; ^4^Department of Biotechnology and Genetic Engineering, Gopalganj Science and Technology University, Gopalganj, Bangladesh; ^5^Department of Clinical Laboratory Sciences, College of Applied Medical Sciences, King Khalid University, Abha, Saudi Arabia; ^6^Pharmacy Discipline, Khulna University, Khulna, Bangladesh

**Keywords:** anticancer, ferulic acid, molecular mechanisms, pharmacokinetics, phenolic substance

## Abstract

Due to the increasing rate of cancer and the undesirable consequences of manufactured drugs, there is a growing interest in the development of natural products as potential remedies. Ferulic acid (FA), a phenolic substance, is found naturally present in the *Ferula foetida* plant's cell walls with therapeutic activities. The objective of this study is to determine the botanical sources, pharmacokinetics, and anticancer activity of FA and its derivatives, focusing on the molecular mechanism by using the data obtained from the literature database. The study's findings suggest that FA demonstrates promising anticancer effects in preclinical pharmacological test methods. The findings of the study exhibit that FA showed promising anticancer activity through underlying mechanisms, including induction of oxidative stress, cytotoxic effect, cell cycle arrest, apoptotic effect, suppression of invasion and migration, antiproliferative effect, autophagy, and genotoxic and mutagenic effect by regulating different molecular pathways like PI3K/AKT, p38/MAPK/ERK, AKT/mTOR, and NF-*κ*B signaling pathways which are involved in cancer development and cell growth. Additionally, this review indicated the pharmacokinetic properties of FA, indicating lower oral bioavailability is affected by the liver's fast conjugation process; this limitation is overcome by applying a nanoformulation of FA. However, additional clinical investigations are recommended to determine the appropriate therapeutic effectiveness, safety, and human dosage.

## 1. Introduction

Cancer is primarily caused by genetic abnormalities resulting in uncontrolled replication and excessive cellular proliferation of normal cells, surpassing the healthy tissue in the surrounding area [1, 2]. After heart disease, cancer is currently the second most common cause of death globally, as reported by the World Health Organization (WHO) [3, 4]. The estimated population of Asia will increase from 6.1 million in 2008 to 10.6 million in 2030 due to aging populations and alterations in lifestyle caused by economic growth in the area [5]. It is projected that there were 20 million incidents of cancer and 10 million deaths directly attributable to cancer across the world. Over the next 20 years, cancer prevalence is expected to rise by 60%, straining health systems, individuals, and communities. By 2040, 30 million additional cancer cases are projected worldwide, and the numbers will expand fastest in countries with low to moderate income levels [6].

Chronic infections can cause cancer, especially in nations with low or middle incomes. The key risk factors for death from cancer globally in low- and middle-income nations were smoking, alcohol consumption, and inadequate consumption of vegetables and fruits. Smoking, drinking alcohol, excessive weight, and obesity were the primary factors contributing to cancer in rich countries [7]. The World Cancer Research Fund and the American Institute for Cancer Research have identified other factors contributing to cancer development. These factors include the consumption of beta carotene, red meat, and processed meats, having a low-fiber diet, not breastfeeding, having a taller adult height, and leading a sedentary lifestyle [8]. Carcinogenic diseases such as *Helicobacter pylori*, hepatitis B, C, HPV, and Epstein–Barr viruses caused 13% of cancers worldwide in 2018. There is presence of cancer in people who also suffer from other significant conditions, like Down syndrome, Parkinson's disease, schizophrenia, diabetes, anorexia nervosa, Alzheimer's disease, allergy-related disorders, and multiple sclerosis [9]. The majority of conditions linked to cancer comorbidity are neuropsychiatric or central nervous system (CNS) disorders [10, 11]. The body's immune system and nervous system send signals from tumor cells to the brain, which may contribute to tumor growth and tumor growth via sympathetic and parasympathetic nerves, hypothalamic–pituitary–adrenal modulation, and adrenal medulla action [12].

Cancer treatment depends on the individual's type, phase, condition, and age. Options for treatment include radiation, surgery, immunotherapy, chemotherapy, and targeted therapy. Early identification and treatment improve survival and minimize difficulties [13, 14]. Multiple challenges of current cancer treatments include resistance, toxicity, low effectiveness, and expense. Anticancer medications can become ineffective due to cancer cell resistance.

Additionally, many anticancer medications have significant adverse effects that affect patients' daily lives [15]. Poor specificity can harm normal cells, causing toxicity and adverse effects [16]. More secure and efficient new anticancer medications are needed [17]. Nanoparticles (NPs) have garnered attention due to their biocompatibility, physicochemical qualities such as large surface-to-volume ratios, conjugation, intriguing functional groups on the surface, and biodegradability in external biological conditions. Through the covalent bonding of cancer receptors in the body, such as antibodies, ligands, immunotoxins, and cytotoxic anticancer drugs, nanobioconjugation is a vital element in the procedure of targeted cancer therapy [18].

Natural compounds have shown potential in treating various kinds of cancer in the drug-creation process [19, 20]. Recently, natural and herbal medications have replaced synthetic chemotherapy medications since they are beneficial to the environment and have few adverse effects. Safety, regulatory approval, and equal efficacy to synthetic photosensitizers are the key obstacles to treating patients using phototoxic and photogenotoxic compounds from plants and herbs [21].

**Figure 1:**
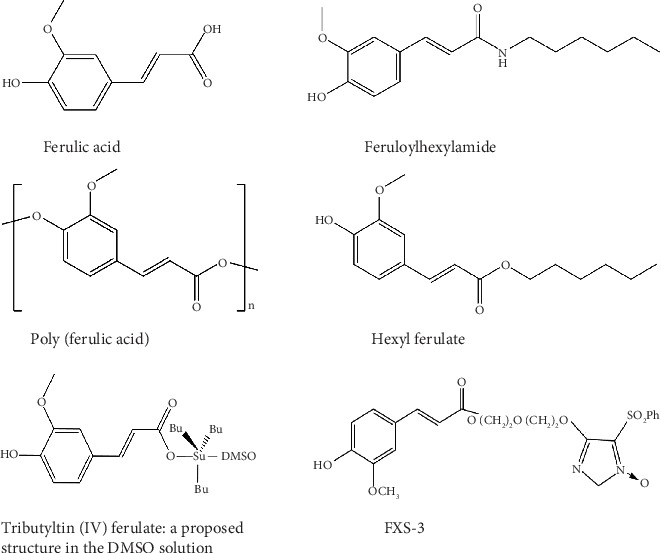
The 2D structures of ferulic acid and its derivatives.

Naturally occurring ferulic acid (FA) (Figure 1), also known as 4-hydroxy-3-methoxy cinnamic acid [22], is a phenolic molecule highly available in *Ferula foetida* plant cell walls [22, 23]. FA displays a diverse range of biological effects, including antioxidant [24], anti-inflammatory [25], antibacterial [26], antiallergic [27], liver-protective [28], anticarcinogenic [29], antithrombotic [30], improving sperm viability, antiviral [31], vasodilatory actions, metal chelation, control of the activity of enzymes, stimulation of transcriptional factors, expression of genes, and signal transduction [22]. FA exerts its effects on various intracellular and extracellular targets and also plays a role in controlling signaling pathways in tumor cells, such as the phosphatidylinositol 3 kinase (PI3K)/protein kinase B (AKT), B cell lymphoma 2 (Bcl-2), and tumor protein 53 (p53) pathways, as well as other signaling routes [32]. FA has been reported to reduce the adverse effects of chemotherapy and radiotherapy in carcinomas by enhancing the innate immune response [22].

The study objective is to provide a complete outline of the current information on the anticancer activities of FA and its derivatives by including diverse studies and findings. In addition, the study highlights the botanical origins, physicochemical characteristics, and pharmacological characteristics of FA.

## 2. Methodology

### 2.1. Data Search

An up-to-date (until December 2024) search was made in the various electronic databases, including PubMed, ScienceDirect, Springer Link, Scopus, Wiley Online, Web of Science, ResearchGate, and Google Scholar, to collect the data. The terms were used for search, including “FA” combined with various terms related to cancer such as “Cancer,” “Tumor,” “Pathophysiology of cancer,” “Apoptotic effect,” “Antiproliferation activity,” “Anticancer activity,” “Oxidative stress,” “Protective effect,” “Carcinogenesis,” “Genotoxic activity,” “Cytotoxic activity,” “Anti-angiogenic effect,” “Anticancer activity,” “Human cancer,” “Biological activities,” “Pharmacokinetics,” “Chemical features,” “Biological evaluation,” “Biopharmaceutics,” “Medicinal use,” “Pharmacology,” “Pharmacological effects,” “Pharmacological activities,” “*In vivo* studies,” or “*In vitro* studies.” There were no limitations on language. The sources, dosage, concentration, test system, predicted therapeutic mechanism, and general conclusions were all included in the extensive evaluation of the studies. More than 100 pieces of evidence were identified through the databases of PubMed and Science Direct, as well as through other sources such as Google Scholar and Research Gate. Based on the inclusion criteria, this study has included 84 reports in which 30 articles described the botanical source and quantities of FA; 43 articles correspond to investigating the anticancer effects of FA in monotherapy, combination therapy, and nanoformulation treatment. Other studies described the clinical evidence and extraction methods of FA from different sources. The criteria for exclusion and inclusion are the following.

### 2.2. Inclusion and Exclusion Criteria

The inclusion criteria include the following: (a) studies conducted focused on animals, humans, and their obtained tissues or cells; (b) studies into the anticancer function of FA and its derivatives; (c) studies involving the integration of FA with additional molecules; (d) studies with or without proposed mechanisms of action; (e) studies exploring both the chemical and physical features of FA; (f) studies examining the biopharmaceutical profiles of FA or its preparations; (g) studies into the toxicological features of FA; (h) clinical studies investigating the effects of FA; and (i) studies investigating the anticancer properties of FA. The exclusion criteria included the following: (i) data, titles, and abstracts that were duplicated and did not meet the requirements for inclusion; (ii) unknown data and evidence that overlapped with other sources; (iii) studies involving FA mixed with additional research; (iv) papers written in languages other than English; (v) studies that lacked complete written content that could be accessed; and (vi) case studies, letters, editorials, and commentaries. The findings are discussed below.

## 3. Biological Sources

Plant tissues contain two types of total FA: conjugate and free [33]. FA is usually found in commelinid plants such as rice (255–362 mg kg^−1^), oats (25–35 mg/100 g) [34], wheat (689 *μ*g/g) [35], pineapple (19.50 mg/100 g) [36], bran of grains such as refined corn bran (8.47 g/kg corn bran) [34], wheat bran (1351–1456 mg/100 g) [33], rye bran (280 mg/100 g), oat bran (33 mg/100 g), peel of fruits like apples (10–1000 ng/mL) [37], banana (219.50 *μ*g/g) [38], grapefruit (18.46 ± 1.65* μ*g/g) [39], and plum (47.87 mg/kg) [40]. Similarly, quantifiable levels of FA were detected in edible portions of 22 types of fruit, eight types of grain, 44 types of vegetables, 14 types of potato, and 25 types of berries [41–44] (Table 1).

## 4. Physicochemical and Pharmacokinetic (PK) Properties of FA

FA contains *trans*-cinnamic acid (including acrylic acid with a 3-position phenyl substituent); at 3 and 4 positions, it bears methoxy and substituents, respectively, on the phenyl ring [31]. The chemical formula of FA is C_10_H_10_O_4_, with a molecular weight of 194.18 g. Its *cis*-form is a yellow liquid, and its *trans-*form is a solid. The average melting temperature of FA is 445.7 ± 1.2 K [67, 68]. The term “bioavailability” describes the proportion and pace at which a drug's initial dosage enters the bloodstream; hence, it is a fundamental component of the PK paradigm. In this instance, a medication's bioavailability is 100% when administered intravenously (IV) [69–71]. The field of PK holds significant importance in drug development as it aids in improving the absorption, distribution, metabolism, and excretion (ADME) characteristics of lead compounds that exhibit favorable in vitro pharmacologic activity [72]. An extensive analysis of main compounds detects any PK problems and toxicity assessment to determine the correct dosage and long-term therapeutic advantages [19, 73, 74]. Thus, research on PK and bioavailability ought to be viewed as crucial elements of drug disposition studies and given careful thought throughout the entire drug development process [70].

Models of *in situ* or *ex vivo* absorption indicate that FA may be absorbed by the stomach [34], jejunum, and ileum [75]. Following a 25-min incubation period, more than 70% of the FA disappeared from the rat stomach and were found in the gastric mucosa, blood, bile, and urine, indicating rapid gastric absorption [34]. FA was also found in the feces of rats around 0.5%–0.8% after ingestion, suggesting a relatively effective rate of FA absorption [76, 77]. The absorbability of FA may indicate its bioavailability to some extent [33]. However, free FA typically has a very low bioavailability because of the liver's quick conjugation process [34]. The relative bioavailability of FA after transdermal administration in rats' plasma was calculated to be 57.9% and in rats' skin 463.4% [78].

Both clinical and preclinical studies demonstrated that FA could be metabolized into two primary metabolites, FA-sulfoglucuronide (60%–90%) and glucuronide (3%–20%), in plasma; also, some other metabolites were found, including FA-diglucuronide, m-hydroxyphenyl propionic acid, feruloyl glycine, dihydroFA, vanillic acid, and vanilloylglycine [34, 76, 79, 80]. The liver is the primary site of FA conjugation [34], the intestinal mucosa [81], and the kidney [82]. FA and its metabolites are eliminated through the bile and urine systems [34, 76, 83]. The short half-life (10–30 min) of FA in rats may indicate its low toxicity, depending on the dose and mode of administration [33, 80].

**Figure 2:**
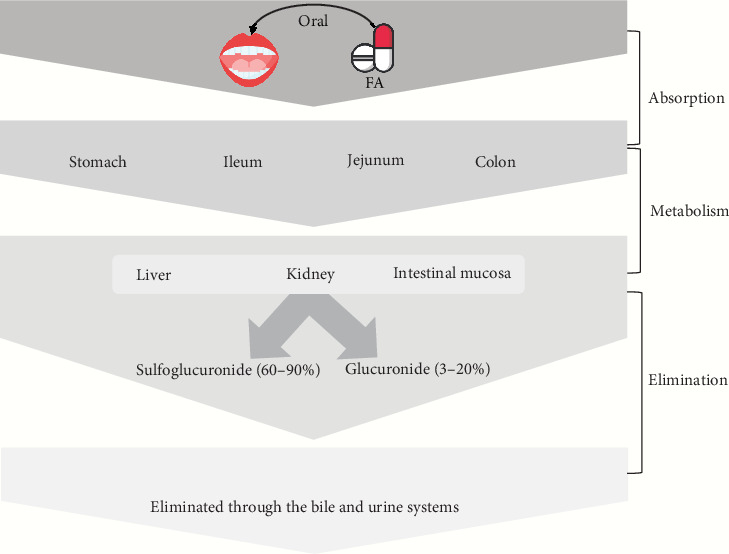
Pharmacokinetics and bioavailability of ferulic acid.

However, due to the poor permeability and solubility of FA, its accessibility to biological systems is inadequate. Its solubility and bioavailability have been attempted to be increased by encasing it in biodegradable polymeric NPs [84, 85]. Drugs and compounds that are sparingly soluble are loaded onto nontoxic chitosan-tripolyphosphate pentasodium (CS-TPP) NPs to increase their bioavailability. FA influences mitochondrial activity to demonstrate its anticancer action because of its enhanced bioavailability [86]. Besides, encapsulation of PLGA NPs (poly lactic-co-glycolic acid nanoparticles) into TFA (*trans*-FA) enhances their solubility as well as bioavailability [87]. The PK activities and bioavailability of FA are displayed in Figure 2.

## 5. Anticancer Activity of FA and Its Derivatives: Underlying Mechanisms

### 5.1. Anticancer Mechanism of FA Through Various Pathways

Cancer is a severe disease that results in deaths globally, with both incidence and mortality rates rising swiftly [88]. Evading apoptosis is recognized as a fundamental property of cancer cells [89]. FA might reduce cancer development through various mechanisms, including modifying the cancer cell cycle, causing apoptosis, cell cycle blockade, and JAK/STAT, NF-*κ*B, PYCR1, PI3K/AKT pathways [90].

In vitro and in vivo studies have demonstrated that FA could effectively impede the spread of colorectal, lung, and breast cancer cells through several mechanisms. Cell cycle is controlled by numerous factors, and disruption of its regulatory mechanisms could result in unchecked proliferation of normal cells and their transformation into tumor cells [91]. Canan et al. reported that FA could impede cell growth by upregulating the gene expression of TP53 and downregulating the gene expression of CDK2, CDK4, and CDK6 in prostate cancer PC-3 cells, resulting in cell cycle arrest in PC-3 cells [32]. In another study, Anwar et al. found that FA could substantially decrease the percentage of cells in S phase, thereby inhibiting the growth of the breast cancer cell line MDA-MB-231 [92]. Nearly every immune regulatory mechanism, both cancer cell identification and tumor-driven immune escape, is controlled by the JAK/STAT3 signaling system. Consequently, JAK/STAT3 pathway inhibitors may suppress tumor cell proliferation and promote antitumor immunity [93]. Guo et al. [94] demonstrated that FA could efficiently lower the phosphorylation level of JAK2/STAT6 in lung cancer A549 cells as well as the levels of expression of the immune factors IL-4, platelet-derived growth factor (PDGF), and granulocyte–macrophage colony-stimulating factor (GM-CSF). This suggests that FA may hinder lung cancer cells from growing and spreading by blocking the JAK2/STAT6 immune signaling pathway [94]. As the NF-*κ*B signaling pathway decreases the production of proapoptotic proteins and activates antiapoptotic genes to promote tumor cell development, it has long been considered a possible target for disease treatment [95]. Maruyama et al. [96] demonstrated that FA could inhibit tyrosinase phosphorylation caused by casein kinase 2 (CK2) in B16 melanoma cells in a dose-dependent manner in vitro, hence preventing NF-*κ*B activation. FA could also decrease tyrosinase activity by directly binding to enzymes [96]. PYCR1 overexpression is linked to the development and growth of cancer [97]. Another study revealed a direct relationship between FA and PYCR1, and FA could dose-dependently suppress the growth of breast cancer MCF-7 and 4T1 cells. By the enzymatic processes, PYCR1 could catalyze proline metabolism and production in vivo, which contributes to the growth and proliferation of tumors. FA has the ability to target PYCR1 and, in a concentration-dependent manner, limit its enzyme activity [98]. The PI3K/AKT/mTOR signaling mechanism regulates cell growth, death, metabolism, and angiogenesis [97]. Luo et al. and their team demonstrated that FA lowered phosphorylation of AKT and PI3K in CaSki cells in a dose-dependent way, resulting in cytotoxicity and death of the cells [99]. In another study, Wu et al. [95] showed that FA reduced the amount of mTOR mRNA and Ki-67 protein in A549 lung cancer graft tissue, increased the levels of caspase-3 protein, and dramatically inhibited tumor growth [95]. However, the study concludes that FA inhibits cancer cell proliferation via several pathways.

### 5.2. Oxidative Stress

Oxidative stress could be defined by a disparity between producing harmful substances called free radicals and reactive compounds, referred to as oxidants or reactive oxygen species (ROS), and removing them by protective mechanisms known as antioxidants [100]. Oxidative stress has been associated with the growth and development of cancer through the amplification of DNA mutations, production of DNA damage, genome instability, and stimulation of cell growth. The growth and spread of tumors and the efficacy of anticancer therapies are greatly affected by the regulation of oxidative stress. ROS metabolism could be regulated by multiple signaling pathways that are linked to cancer through either direct or indirect processes [101, 102]. For example, the suppression of the MAP kinase phosphatase by ROS leads to the stimulation of ERKs. ROS-induced reduction of PTEN leads to the hyperactivation of the PI3K/AKT signaling pathway, which is often seen in cancer cells [103, 104]. NF-*κ*B is also a significant mechanism that regulates the equilibrium of the cellular oxidative condition [105]. So, the control of oxidative stress could be an essential approach to cancer treatment.

A study found that FA decreased the longevity of HeLa and CaSki cells in vitro at concentrations ranging from 4 to 20 *μ*M by promoting DNA reduction and apoptosis. Moreover, treatment with FA elevated pro-caspase-3, pro-caspase-8, and pro-caspase-9 and PARP cleavage, Bax, and ROS and decreased Bcl-2, Mcl-1, AKT, and PI3K pathway levels in a dose-dependent way [99]. Another investigation showed that FA induced antioxidant activity in lung cancer A549 cells and colon cancer HT29-D4 cells at a concentration of 50–1000 *μ*M by reducing the superoxide production [106]. Somade et al. also demonstrated that FA (25 and 50 mg/kg) showed antioxidant activity in male Wistar rats in hepatocellular carcinoma via increasing p53 and Nrf2 and decreasing NF-*κ*B, superoxide dismutase (SOD), and CAT [107]. Furthermore, a study also suggested that FA showed antioxidant activity in esophageal cancer cells (TE-4 and EC-1) at a concentration of 20−60 *μ*M by upregulating ROS and downregulating SOD production [108]. Fong et al. revealed that TFAs showed potent antioxidant characteristics by increasing ROS levels in H1299 cells, including hydrogen peroxide and superoxide anion in lung cancer [109]. These findings revealed that FA could destroy cancer cells by inducing oxidative stress through different mechanisms.

### 5.3. Cytotoxic Effect

Cytotoxicity is the primary mechanism by which chemotherapeutic medicines induce cell damage. Assessing the toxicity of a substance on cells is an essential step in the advancement of medications used for the management of cancer [110, 111]. The cytotoxic effect of a chemical substance significantly improves its potential as a chemotherapy drug. These drugs have been developed to preferentially focus on and destroy cancer cells that undergo rapid division to impede the growth and advancement of tumors [112, 113]. Evaluations of the cytotoxic effects on cells, which rely on time and concentration, are commonly employed in testing for anticancer drugs. Cytotoxic medicines include the ability to disrupt the process of DNA synthesis or cause chemical destruction of DNA, leading to the eventual death of cancerous cells [114]. So, phytochemical compounds that exhibit notable cytotoxic effects have the potential to be evaluated for further research in the production of anticancer drugs.

Several studies have shown the cytotoxic properties of FA on different types of cancer cells, suggesting its potential as an option for additional research into its anticancer effects. In addition, FA has a cytotoxic effect against 4T1 breast cancer cells [115]. FA has a prompting cytotoxic effect against breast cancer MDA-MB-231 in vitro and female BALB/c nude mice [116]. Another research revealed that FA derivative feruloylhexyl-amide (HFA) also showed a cytotoxic effect against breast cancer cells (MCF-7, MDA-MB-231, and HS578T) [117]. Research suggested that FA showed cytotoxic effects against cervical cancer HeLa and CaSki cells [99]. Furthermore, Roy et al. demonstrated that FA has a significant cytotoxic effect against colorectal cancer HCT 15 cells with an IC_50_ value of 154 *μ*g/mL [118]. According to Chen et al. [119], FA (800 *μ*M) induced cytotoxicity against CT-26 cells in colon cancer with an IC_50_ value of 800 *μ*M [119]. A study revealed that tributyltin (IV) ferulate is the derivative of FA and has a cytotoxic effect against HCT116, Caco-2, and HT-29 cells in colon cancer [120]. Another study revealed that FA showed promising cytotoxic effects against the pancreatic cancer MIA PaCa-2 cell with an IC_50_ value of 500 *μ*M/mL [121]. FA (100–200 *μ*g/mL) showed promising cytotoxicity against MCF-7 and HepG2 cell lines in both breast cancer and liver cancer [122]. Cao et al. demonstrated that FA (20−60 *μ*M) exhibits cytotoxicity against TE-4 and EC-1 cells in esophageal cancer [108]. A study demonstrated that FXS-3, which is the derivative of FA, has a promising cytotoxic effect against lung cancer A549 cells in male BALB/C nude mice with an IC_50_ value of 50 *μ*M [123]. Sucu and Koc revealed that FA induced cytotoxicity in LN229, T98G, and U87 cells in glioblastoma [124]. Another study strongly evidenced that FA exhibits cytotoxic effects against breast cancer cells (MCF-7 and MDA-MB-231), lung cancer cells (A549), liver cancer cells (HepG2), and cervical cancer cells (HeLa) [125].

### 5.4. Cell Cycle Arrest

Cell division from one parent cell results from a sequence of coordinated processes called the cell cycle. Various factors control the cell cycle, and an imbalance in the regulatory system could result in the uncontrolled development of normal cells and their conversion into tumor cells [91]. Several mechanisms and pathways also control the cell cycle. By regulating this mechanism and pathways, cancer cell proliferation can be inhibited. As a result, this mechanism is used by potential anticancer drugs in cancer therapy [126]. Specific proteins are targeted with various chemotherapy drugs to inhibit cell growth through different cell cycle phases, resulting in an accumulation of cancer cells at specific points in the cycle. The cell cycle halts, inhibiting the cancer cell's ability to undergo rapid division and form tumors, as well as blocking its spread to other regions of the body [127].

Several studies revealed that FA exhibits anticancer effects by arresting the G0/G1/S phases of the cell cycle. Yue et al. found that A549 cells were treated with FXS-3 at concentrations 0.2–50 *μ*M in vitro, and male BALB/C nude mice were treated with FXS-3 at concentrations 25–100 mg/kg in vivo. This treatment blocked the cell cycle at the G0/G1 phase by downregulation of JNK, AKT/mTOR, and MEK/ERK pathways [123]. Another study showed the anticancer activity of FA, including its ability to arrest the cell cycle in colon cancer. Results showed that treating with Caco-2 cells at a concentration of 1500 *μ*M remarkably enhanced the length of the S phase and cell cycle arrest at the S phase [128]. Gao et al. showed that FA was tested on Hela and CaSki cervical carcinoma cell lines at concentrations from 4 *μ*M, which led to cell cycle arrest in the G0/G1 phase by reducing cell invasion, MMP9 mRNA expression, and cyclin D1 and cyclin E levels [129]. Another investigation found that the tributyltin ferulate derivative of FA was tested on HCT116, HT-29, and Caco-2 colon cancer cells at a concentration of 40 *μ*M which stopped the G0/G1 phase of the cell cycle, leading to a reduction in RIPK1 and an elevating in cytotoxicity, ultimately causing the death of the cells [120].

### 5.5. Apoptotic Effect

Apoptosis, also known as programmed cell death, is a mechanism that can occur through two distinct methods. The initial process, known as the extrinsic pathway, is initiated by the stimulation of caspase-8 and caspase-10 through the creation of DISC (death-inducing signaling complex). This activation is triggered by the stimulation of tumor necrosis factor (TNF) or FAS ligand. The second pathway, the intrinsic pathway, is started by activating the mitochondrial outer membrane permeabilization. This activation is triggered by cytochrome c and another activator of caspase that originates from the mitochondria [130]. The intrinsic pathway could be activated by multiple variables, such as damaged DNA caused by exposure to pharmacological, chemical, or oxidative stress. This leads to an elevated level of the synthesis of the tumor-suppressing protein p53, which in turn triggers apoptosis by elevating the concentration of Bax and decreasing the concentration of Bcl-2 protein simultaneously [131, 132].

Research findings demonstrated that FA at concentration 3–100 *μ*M MDA-MB-231 cell in vitro and MDA-MB-231 xenograft mouse model in female BALB/c nude mice in vivo (*n* = 6) at concentration 100 mg/kg could induce apoptosis by increasing cytotoxicity and decreasing tumor volume and weight, EMT process, and cell growth [116]. Another study revealed that FA (4–20 *μ*M) could stimulate apoptosis in HeLa and CaSki cells by increasing cytotoxicity, pro-caspase-3, pro-caspase-8, and pro-caspase-9, PARP, Bax, ROS, DNA condensation, and decreasing Bcl-2 and Mcl-1, AKT, PI3K phosphorylation in cervical cancer [99]. A study by Chen et al. [119] also found that FA extensively plays a vital role in inducing autophagy and apoptosis of colon cancer CT-26 cells at a concentration of 800 *μ*M and BALB/c mice via increasing ERK, JNK, cytotoxicity, Bax and decreasing Bcl-2, NF-*κ*B, and TNF-*α* [119]. Alazzouni et al. showed that FA (50 mg/kg) could induce apoptosis in adult male Wistar albino rats in colon cancer by elevating p53, caspase-3, Ki67, and CK20 [133]. Another investigation revealed that FA could induce apoptosis and autophagy in HCT116, Caco-2, and HT29 cells in vitro at a concentration of 100–400 nM in tributyltin (IV) ferulate in colon cancer by G2/M cell cycle arrest and downregulate RIPK1 levels [120]. FA increased Bax, caspase-3, and decreased Bcl-2 and PI3K/AKT pathway in143B and MG63 osteosarcoma cells which are the indicators of apoptosis [134]. A study demonstrated that FA could trigger apoptosis by elevating the caspase-3 protein, Bax, and reducing procaspase-3 protein, Bcl-2, in SaOS-2 and MG63 cell lines at 40-*μ*M doses in osteosarcoma cancer [135]. Another study found that FA stimulated apoptosis by increasing the expressions of ATR, RB1, and TP53, as well as reducing the gene expressions of CCND1, CCND2, and CCND3 in PC-3 cells in prostate cancer cell lines [32]. In breast and liver cancer cells (MCF-7 and HepG2), FA enhanced apoptosis via upregulating caspase-8 and caspase-9 [122]. In the context of hepatocellular carcinoma cells (HepG2), it has been shown that FA (100 *μ*g/mL) enhanced apoptosis via increasing PINK-1, Parkin and reducing the MMP expression [136]. Furthermore, a study demonstrated that FA induced programmed cell death in esophageal cancer cells (TE-4 and EC-1) by increasing ROS, cytotoxicity, LDH release, and caspase-3 while decreasing SOD cell growth [108]. FA could induce cell death of cell lung cancer cells (H1299) at a concentration of 0.06–0.6 *μ*M by upregulating ROS, hydrogen peroxide, superoxide anion, and downregulating MMP2 and MMP9, colony formation, and AIG capacity [109]. It also found that FA derivative FXS-3 showed anticancer activity that resulted in enhanced death of lung cancer A549 cells via negatively controlling ERK/p38, AKT/mTOR, and MEK/ERK signaling pathways, which provides the crucial scientific foundation for the advancement of anticancer medications involving FA derivatives [123]. This finding shows evidence that FA has promising therapeutic effects in both the management and treatment of cancer.

### 5.6. Inhibition of Invasion and Migration

Tumor invasion and metastasis refer to the phenomenon where tumor cells invade and spread from the initial lesion to adjacent tissues, ultimately enrolling adjacent tissues or potentially spreading throughout the entirety of the body [137]. The proliferation of malignant cells creates a significant risk to human life since they possess the ability to spread from the impacted organ to adjacent healthy tissue by utilizing the blood and lymphatic systems as primary routes for migration [138]. The fundamental mechanism of migration is characterized by the dynamic coupling and interaction with the tissue that surrounds the structure [139]. In addition, there are different pathways, such as the STAT3, NF-*κ*B, and AKT/GSK 3*β*/Snail signaling pathways, that play an essential role in the migration and invasion process associated with metastasis [140]. NF-*κ*B activity has the impact of enhancing the development of cancer cells, inhibiting cell death, and promoting the creation of new blood vessels by triggering a process called epithelial–mesenchymal transition, which in turn accelerates the spread of cancer to distant parts of the body. Activation of NF-*κ*B can also alter the local metabolism and suppress the immune system to facilitate the growth of tumors. The NF-*κ*B pathway is a possible target for therapy due to its ability to induce tumor regression when inhibited in myeloid cells or tumor cells [141–143].

Research findings revealed that the phytochemical FA suppressed the cancerous cell migration and invasion to other organs. A study demonstrated that FA could suppress the migration of the MDA-MB-231 cell line in breast cancer by decreasing cell proliferation metastasis and affecting the EMT process [116]. Another investigation showed that FA (4 *μ*M) could suppress migration of Hela and CaSki cervical carcinoma cell lines by increasing the cell cycle arrest in the G0/G1 phase and decreasing MMP9, mRNA expression, and cyclin D1 and cyclin E levels [129]. FA suppressed the migration and invasion effect of pancreatic cancer cells (MIA PaCa-2) in vitro, resulting in upregulating p53, Bax, caspase-3, and caspase-9 and downregulating the Bcl-2, CCND1, and CDK 4/6 level [121]. In addition, it has been found that FA (200 *μ*M) suppressed the invasion of lung cancer A549 cells by decreasing superoxide production, cell adhesion, and proliferation [106]. Eroğlu et al. show that FA plays a crucial role in the case of reducing the invasion of prostate cancer (PC-3) in vitro at a concentration of 20 *μ*M–2 mM by increasing expressions of ATR, ATM, CDKN1A, CDKN1B, E2F4, RB1, and TP53 and reducing the gene expressions of CCND1, CCND2, CCND3, CDK2, CDK4, and CDK6 level in PC-3 cells [32]. Furthermore, a study demonstrated that FA could inhibit the migration of esophageal cancer cells (TE-4 and EC-1) by increasing ROS, cytotoxicity, LDH release, and caspase-3 and decreasing SOD, cell growth, colony formation, and angiogenesis [108]. Guo et al. demonstrated that FA could inhibit the migration of A549 lung cancer cells via increasing p53 and decreasing IL-4, PDGF, P-JAK2, P-STAT6, XIAP, and COX-2 expressions [94]. Fong et al. [109] demonstrated that TFA (0.06–0.6 mM) inhibits the migration of lung cancer H1299 cells by decreasing the expression of MMP2 as well as MMP9 protein [109]. Another study revealed that FA derivative (FXS-3) also plays a vital role in the inhibition of migration and invasion of lung cancer A549 cells (0.2–50 *μ*M) and male BALB/C nude mice (25, 50, and 100 mg/kg) [123].

### 5.7. Autophagy

Autophagy is a process occurring in cells involving the breakdown and recycling of intracellular components. It depends on lysosomes, which degrade dysfunctional or old organelles [144]. It has a vital role in cancer, including its preventive benefits against cancer formation and potential function in advancing the spread of cancer [145, 146]. Several investigations demonstrate that the signaling transducer and activator of transcription 3 (STAT3) influences autophagy by stimulating the antiapoptotic Bcl-2 family, regulating autophagy through the Bcl-2–Beclin-1 complex. Beclin-1 is a crucial protein that triggers autophagy and contains a BH3 domain that could bind to antiapoptotic Bcl-2-related molecules [147, 148]. Furthermore, p62, mTOR, and AMPK as well as autophagy-ATG genes have been playing an essential role in conserving and controlling autophagy [149, 150]. Therefore, under specific conditions, targeted pharmacological control of autophagy carries significant possibilities as an innovative therapeutic strategy, thus expanding the present array of cancer treatments [136].

Numerous studies have demonstrated that FA has shown anticancer activity via the increase and decrease of autophagy in different types of cancer. FA at concentrations of 4 *μ*M in both Hela and CaSki cell lines, in vitro, resulted in inhibiting autophagy in cervical cancer. This effect was achieved via the stimulation of p53 and p21 and suppression of LC3-II, Beclin-1, and Atg12–Atg5 [129]. Similarly, Chen et al. reported that FA enhanced autophagy in CT-26 cells, at concentrations of 800 *μ*M and BALB/c mice, in vivo (*n* = 8) at concentrations of 40 and 80 mg/kg in colon cancer cells by upregulating ERK, JNK, and Bax and downregulating NF-*κ*B, TNF-*α*, and IL-1*β* expression [119]. Pellerito et al. [120] showed that FA induced autophagy in HCT116, Caco-2, and HT29 cells, in vitro, at 100–400 nM dose of tributyltin in colon cancer via increasing G2/M cell cycle arrest, cell death, LC3-II, and p62 and decreasing the RIPK1 expression [120]. In the context of hepatocellular carcinoma cell (HepG2), it has been shown that FA (100 *μ*g/mL) enhanced autophagy, with increasing Beclin-1, LC3-I/LC3-II, PINK-1, and Parkin and decreasing MMP expression [136].

### 5.8. Antiproliferative Effect

Proliferation is a circadian process that is triggered by numerous factors, such as disruption of hormone feedback pathways, tissue trophic activity inhibition, persistent antigenic material presence, persistent cytotoxicity, and cell death [151]. The spread and growth of cancer are greatly influenced by the alteration of protein expression and function linked with the cell cycle. Continuous stimulation of many signaling mechanisms also facilitates cellular growth and maturation [152]. Numerous signaling pathways, including EGFR-ERK1/2, MAPK, PI3K, and mTOR, play a crucial role in controlling cell proliferation, growth, and survival, as well as interrupted in various forms of cancer [153, 154]. Abnormal mTOR activation happens frequently in cancer and is an essential phase in the advancement of the disease [155]. Inhibiting these mechanisms is an appropriate approach for the improvement of anticancer medication. Within this context, numerous studies demonstrated that FA suppresses cancer cell proliferation and tumor development in various types of cancers.

**Figure 3:**
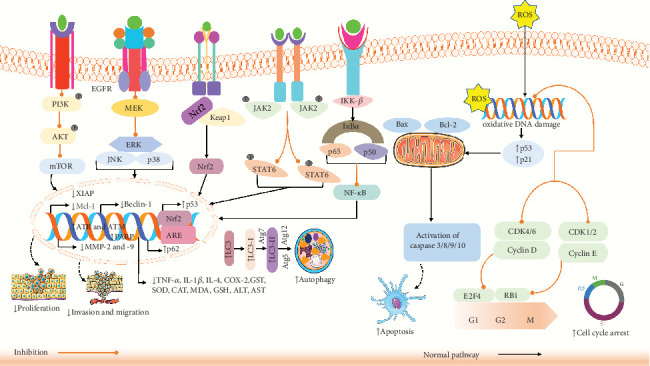
Estimated possible anticancer mechanism of FA involving various cellular pathways. MMP9: matrix metalloproteinase-9; caspase: cysteine-aspartic acid protease; PARP: poly-ADP ribose polymerase; Bax: Bcl-2-associated X; ROS: reactive oxygen species; Bcl-2: B cell lymphoma protein 2; Mcl-1: myeloid cell leukemia 1; AKT: protein kinase B; PI3K: phosphatidylinositol-3 kinase; ERK: extracellular signal–related kinases; JNK: c-Jun NH2-terminal kinase; NF-*κ*B: nuclear factor kappa-light-chain-enhancer of activated B cells; p53: tumor protein p53; CCND1: cyclin D1; CDK: cyclin-dependent kinase; Nrf2: nuclear factor erythroid 2–related factor 2; GST: glutathione S-transferases; SOD: superoxide dismutase; JAK2: Janus kinase 2; STAT6: signal transducer and activator of transcription 6; XIAP: X-linked inhibitor of apoptosis protein; MEK: mitogen-activated extracellular signal–regulated kinase; LC3-II: microtubule-associated protein 1A/1B-light chain 3; p62: ubiquitin-binding protein p62; mTOR: mammalian (or mechanistic) target of rapamycin; Beclin-1: protein which regulated autophagy; IL-2; Atg 5, 7, and 12: autophagy protein 5, 7, and 12; p65: transcription factor p65; p38: mitogen-activated protein kinase; p50: transcription factors; p21: inhibitor of cyclin-dependent kinase; STAT3: signal transducer and activator of transcription 3; Keap1: Kelch-like ECH-associated protein 1; IKK-*β*: inhibitor of nuclear factor kappa-B kinase subunit beta.

A study by Bagheri et al. showed that FA (500 *μ*g/mL) enhanced antiproliferative activity in the 4T1 cell line in breast cancer [115]. Another study revealed that FA at concentration 3–100 *μ*M MDA-MB-231 cell in vitro and MDA-MB-231 xenograft mouse model in female BALB/c nude mice in vivo (*n* = 6) at concentration 100 mg/kg could suppress the metastasis of cancer cell by stimulating apoptosis and inhibiting EMT process [116]. Serafim et al. showed that FA at a concentration of 25–75 *μ*M could suppress the proliferation of breast cancer cells (MCF-7, MDA-MB-231, and HS578T) [117]. Another investigation showed that FA (4 *μ*M) could upregulate the antiproliferative activity in Hela and CaSki cervical carcinoma cell lines [129]. Furthermore, another study of FA (100–250 *μ*g/mL) showed inhibition of proliferation in colorectal cancer cells (HCT 15) through downregulation of epidermal growth factor receptor (EGFR) [118]. Chen et al. [119] revealed that FA significantly plays a vital role in reducing the proliferation of colon cancer cells (CT-26) via inducing apoptosis and ERK, JNK pathway [119]. Alazzouni et al. showed that FA could inhibit the metastasis of colon cancer in adult male Wistar albino rats at a concentration of 50 mg/kg by increasing apoptosis and Ki67 protein expression [133]. A study found that FA plays a vital role in inducing antiproliferative activity of colon cancer in Caco-2 cells at a concentration of 150 *μ*M through increasing RABGAP1, CEP2, and SMC1L1 expression [156]. In another study, FA (1500 *μ*M) elevated antiproliferative effects on colon cancer cells (Caco2) by impacting many stages of the cell cycle, explicitly causing the S phase to become longer, which in turn inhibits cell proliferation [128]. Bouzaiene et al. showed that FA could induce antiproliferative activity at a concentration of 50–1000 *μ*M in A549 cells in lung cancer and HT29-D4 cells in colon cancer in vitro by reducing superoxide production [106]. A study also showed that FA could reduce proliferation in 143B and MG63 osteosarcoma cells in vitro at a concentration of 10–150 *μ*M and MG63 xenograft model in mice in vivo at a concentration of 100 mg/kg by inducing apoptosis and reducing CDK 2, 4, and 6, PI3K/AKT pathway [134]. A study by Eroğlu et al. [32] found antiproliferative action of FA in prostate cancer cells (PC-3 and LNCaP cells), which showed by elevating the gene expressions of ATR, RB1, ATM, CDKN1A, TP53, E2F4, and CDKN1B and reducing the gene expressions of CCND1, CDK2, CCND2, CCND3, CDK4, and CDK6 in PC-3 cells [32]. Wang et al. [136] found that cell proliferation was suppressed in HepG cells and in vitro hepatocellular carcinoma by increasing apoptosis and LC3-I/LC3-II, PINK-1, Parkin and decreasing MMP expression with FA (100 *μ*g/mL) treatment [136]. Guo et al. demonstrated that FA could suppress the spread of A549 lung cancer cells via decreasing IL-4, PDGF, P-JAK2, P-STAT6, XIAP, and COX-2 expression [94]. Another investigation found that FA could hinder the proliferation of H1299 cells, cell viability, and proliferation assay in vitro at a concentration of 0.06–0.6 *μ*M of TFA in lung cancer by upregulating ROS, hydrogen peroxide, and superoxide anion and downregulating MMP2 and MMP9 [109]. It also found that FA derivative FXS-3 showed inhibition in proliferation of lung cancer A549 cells in vitro and xenograft mouse model in male BALB/C nude mice (A549 cells) in vivo (*n* = 8) by promoting JNK signaling pathway and negatively controlling ERK/p38, AKT/mTOR, and MEK/ERK signaling pathways, which showed essential scientific foundation for the development of anticancer medication about FA derivatives [123]. The overall possible anticancer mechanisms of FA and its derivatives are illustrated in Figure 3 and Table 2.

### 5.9. Genotoxic and Mutagenic Effects

Genotoxicity refers to the capacity of toxic agents to cause damage to genetic material. It is commonly mistaken for the mutagenic impact, which specifically relates to the permanent changes in the quantity and composition of genetic materials in cells or animals that might lead to increased mutations. Genotoxicity includes mutagenicity, although not all genotoxic agents are mutagenic as they may not induce genetic changes in the sequence of DNA [157, 158]. It is widely recognized that multiple significant DNA repair pathways are involved in the process of repairing DNA damage caused by pollutants that ultimately result in alterations in genes [159]. The PI3K/AKT/mTOR pathway is essential for cancer cell motility, proliferation, survival, and metabolism. Consequently, it is recognized as one of the most commonly compromised mechanisms in cancer, resulting in a prospective strategy for therapeutic intervention [160, 161]. Anticancer medications could also function by inducing genotoxic and mutagenic impacts on cancer cells [110].

Luo et al. [99] expressed that FA induced DNA damage in HeLa and CaSki cells in cervical cancer via increasing PARP, Bax, and ROS and decreasing AKT, PI3K phosphorylation. This finding indicates that FA could be a promising genotoxic and mutagenic potential for cancer management [99].

### 5.10. Anticancer Activity of FA in Combination With Other Molecules

The approach of combining two or more therapeutic compounds is the basic principle of combination therapy. The aggregation of different natural compounds increases the efficacy of anticancer activities in contrast to the single therapeutic methodology due to the effectiveness of targeting major pathways in a synergistic way [162]. The use of combinations of molecular-targeted agents (MATs) has been recommended as a method to overcome the limitations of shutting down a single target in eradicating cancer, and some pioneering research has been carried out by researchers to determine the effectiveness of this approach [36]. Combination therapy has excellent efficacy in both hormonal and targeted therapies, which can also prevent alternate treatment-resistant pathways. Moreover, combination therapies can increase treatment effectiveness by decreasing side effects related to single-drug therapy, such as induced metastasis [163]. FA could induce efficacy of anticancer treatment via inducing apoptosis, cytotoxicity, and cell death and reducing cell proliferation and cell viability by combining with other compounds like 2-deoxy-D-glucose (2DG) [164], epirubicin, gamma radiation [165], aspirin, thyoquinine [166], phenolic and flavonoids, P-coumaric acid [167], 4-vinylguaiacol, caffeic [168], coumaric, and gemcitabine [169].

**Figure 4:**
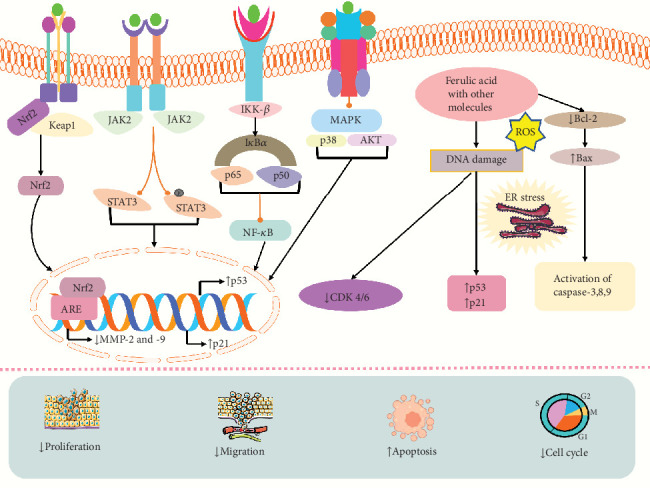
Possible anticancer mechanism of FA in combination therapy. Bax: Bcl-2-associated X protein; Bcl-2: B cell lymphoma; ROS: reactive oxygen species; MMP2: matrix metalloproteinase-2, 9; MAPK: mitogen-activated protein kinase; AKT: protein kinase B; p65: transcription factor p65; AKT: protein kinase B; Nrf2: nuclear factor erythroid 2–related factor 2; p38: mitogen-activated protein kinase; p50: NF-*κ*B family transcription factors; p53: tumor protein P53; p21: inhibitor of cyclin-dependent kinase; STAT3: signal transducer and activator of transcription 3; JAK2: Janus kinase 2; Keap1: Kelch-like ECH-associated protein 1; IKK-*β*: inhibitor of nuclear factor kappa-B kinase subunit beta; NF-*κ*B: nuclear factor kappa B; caspase: cysteine-aspartic acid protease.

A study by Bandugula and Rajendra Prasad showed that the combination of 2DG and FA could induce efficacy of anticancer activity by increasing the expression of p21, p53, Bax, GADD45A, and caspase-3, which caused cell death, cytotoxicity, and cell cycle arrest in lung cancer cells (NCI-H460) and also suppressed the expression of NF-*κ*B [166]. Another study found the anticancer activity of FA and epirubicin demonstrated that they could trigger apoptosis via upregulating the expression of Bax, caspase-3, and ER stress-related protein and downregulating Bcl-2 and cell proliferation in a breast cancer cell line (MDA-MB-231) at the dose of FA (10 and 100 *μ*M) and epirubicin (0.02–4 *μ*M) [165]. FA and gamma radiation could induce radiation effects, TBARS, CD, and LHP, levels of lipid peroxidation, ROS levels, oxidative DNA damage, and cytotoxicity and diminish the number of colonies, survival rate in cervical cancer cells (HeLa and ME-180) at 1–40 *μ*g/mL concentrations [170]. An in vitro and in vivo study by Thakkar et al. [169] revealed that FA and aspirin could trigger apoptotic cell death, p-RB, p21, and p-ERK1/2, cytotoxicity and reduce PCNA and MKI67, growth of tumor in pancreatic cancer. FA and thyoquinine showed anticancer activity in breast cancer by increasing apoptosis and decreasing cell proliferation [92]. A recent study by Salam et al. demonstrated that FA could inhibit breast and colon cancer by combining with some phenolic and flavonoids by triggering cytotoxicity, cell death, Bax, caspase-9, and p53 and reducing cell proliferation and Bcl-2 expressions [168]. In colorectal cancer cells (HCT-116 HT-29 and SW620), FA and P-coumaric acid could decrease PKM2, cell growth, aerobic glycolysis, and ATP production [171]. Similarly, FA and 4-vinylguaiacol could induce apoptosis, caspase-3, caspase-8, and caspase-9 cell death and reduce proliferation cell growth in colorectal cancer cells [167]. The combination of FA with caffeic coumaric caused cytotoxicity and apoptosis by reducing the expression of the Htert gene in hepatocellular carcinoma cells (HepG2) at 25−800 *μ*g/mL doses [164]. The anticancer activities of a combination of FA with ligustrazine and tetrahydropalmatine are investigated in endometrial cancer cells (Hem15A and HEC1-B) by downregulating MMP2 and MMP9, invasion, metastasis, ectopic volume, and cell growth and inducing TIMP-1 [172]. FA and IR combination treatment in lung and liver cancer cells (A549 and HepG2) showed that they could stimulate ROS level, Keap1 level, cytotoxicity, mitochondrial apoptosis, p53, p21, G2/M phase arrest, cyclin B1, and mitotic arrest and diminish cell proliferation, p-AKT/pp38MAPK, NF-Jb/p65, Nrf2, MMP9, VEGF, STAT3, Nrf2, the cell cycle progression, Cox-2, and Cdc25C at the dose of 10–400 *μ*M [173]. FA and gemcitabine could stimulate death in prostate cancer cells (PC-3) [174] (Table 3). The mechanism of action of FA in combination therapy is depicted in Figure 4.

### 5.11. Anticancer Activity of FA-Loaded Nanoformulations

Scientists have been using nanomaterials, which exhibit unique optical, magnetic, and electrical characteristics as a result of their size range of 1–100 nm. FA-loaded nanoformulations have been developed by using different nanomaterials to improve cancer treatment by reducing the harmful effects on cells, increasing the effectiveness of the treatment, raising the amount of medicine that could be delivered, and enhancing the availability of the drug in the body [175]. NP-based medication delivery offers numerous benefits over conventional drugs, including higher stability and compliance, increased permeability, and retention effect, with precision targeting [176]. The combination of plant-based medicine with the use of nanotechnology in medical practice holds the capacity to enhance the efficacy of medications and result in enhanced outcomes for patients [177].

**Figure 5:**
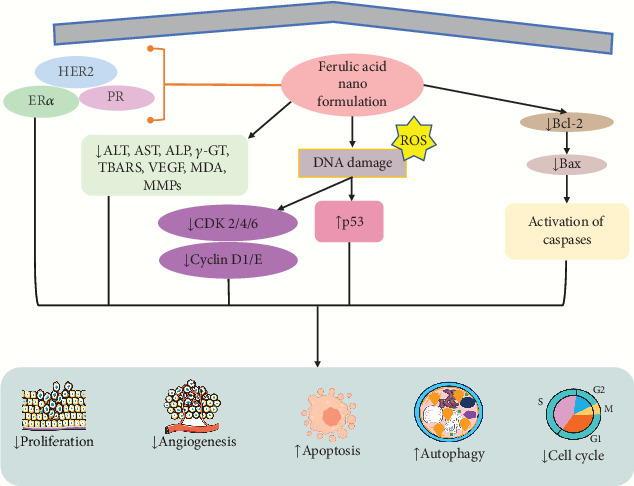
Mechanism of action of ferulic acid–loaded nanoparticles. HER2: human epidermal growth factor receptor 2; ER*α*: estrogen receptor *α*; PR: progesterone receptor; Bax: BcL2-associated X protein; p53: tumor protein p53; Bcl-2: B cell lymphoma; ROS: reactive oxygen species; ALT: alanine transaminase; AST: aspartate aminotransferase; ALP: alkaline phosphatase; TBARS: thiobarbituric acid reactive substance; MMP: matrix metalloproteinase; VEGF: vascular endothelial growth factor; *γ*-GT: gamma-glutamyl transferase; CDK2/4/6: cyclin-dependent kinase 2/4/6.

A finding demonstrated that the nanoformulation of FA-NS (1:4) induced cytotoxicity against MCF7 and 4T1 cells in breast cancer revealed via different concentrations (5–1000 *μ*M) with IC_50_ value of 250 *μ*M via reduced cell proliferation and enhanced lipid peroxidation and apoptosis of cancer cells [178]. Another study revealed that FA-encapsulated electrospun PLGA/PEO nanofibers at optimum 2 wt% concentration showed an apoptotic effect in breast cancer cells (MCF-7 and HEK-293) by MTT assay in vitro with reducing proliferative activities [179]. Panwar et al. [86] showed that FA in CS-TPP NPs boosts its efficacy as a medicinal drug. FA/CS-TPP NPs against ME-180 cervical cancer cells show more significant cytotoxicity compared to native FA with an IC_50_ value of 40 *μ*M by inducing apoptosis and cytocompatibility of cells [86]. Zheng et al. [180] revealed that the evaluation of CT26 cells in a male BALB/c mouse model with colon cancer suggested that PFA NPs have a drug-loading capacity of around 8.3% of paclitaxel (PTX), resulting in the induction of apoptosis, lowering both the proliferation and tumor growth in vitro and in vivo [180]. Furthermore, the conjugation of ZnONPs with FA showed anticancer potential in colon cancer cells (Huh-7 and HepG2) at a dose of FA (0.05–50 *μ*g/mL) and ZnONPs (10–150 *μ*g/mL) with IC_50_ value of 8.2 *μ*g/mL resulting in induced of apoptosis via increasing ROS, DNA damage, autophagy, and cell cycle arrest in S-phase, as well as cytotoxicity of cells and decreasing the ALT, AST, ALP, *γ*-GT, and TBARS level [122]. Another study revealed that the nanoformulation of Dox/FA-PLGA-TFA NPs showed anticancer activity in breast cancer in female Sprague–Dawley rats in vivo (*n* = 8) at a dose of 40 and 80 mg/kg concentration by reducing P-gp level, Notch1, Hes1, Wnt-3a, *β*-catenin, MMP9, cyclin D1, ER*α*, PR, HER2, and serum ALT [87]. Kumar et al. demonstrated that chitosan-coated trans-resveratrol (RSV) and FA (FER)–loaded SLNs showed anticancer effect in in vitro studies using HT-29 cells resulting in induced apoptosis by increasing G0/G1 phase cell cycle arrest, Bax, and p53 and reducing Cyclin D1 and E, CDK 2, 4, and 6, MMP, and Bcl-2 [181]. Another investigation showed that FA-loaded polymeric and lipidic NCs were evaluated on colorectal cancer cell lines (HCT-116 and Caco2 cells) in vitro and also tested in vivo on rats. The study exhibited that the NCs exhibited anticancer and anti-inflammatory effects compared to the drug alone with induction of apoptosis evidenced through increasing Bax and decreasing Bcl-2, MDA, IGF II, VEGF, and cyclin D1 in both cell lines [182] (Table 4). The mechanism of action of FA-loaded NPs is shown in Figure 5.

## 6. Clinical Evidence

Polyphenolic compounds also known as flavonoids have the potential to be used as therapeutic agents. Different investigations have revealed the stronger anticancer potential of these natural products [183]. There is an absence of clinical data for the use of FA in the diagnosis of cancer. However, different studies have demonstrated the effect of FA supplements on various diseases [184–186]. A clinical study conducted by Bumrungpert et al. revealed that FA could induce the level of HDL-C and reduce BAP, cholesterol, LDL-C, triglyceride, oxidative stress biomarker, MDA, LDL-C, inflammatory markers hs-CRP, and TNF-*α* in hyperlipidemia patients. They used 1000 mg of FA daily in 48 hyperlipidemic subjects between 20 and 60 years old [184]. Another clinical study demonstrated the beneficial effect of FA on skin barrier function by upregulating stratum corneum hydration and enhancing the strength of SBF in healthy men. FA also diminished *trans*-epidermal water loss and the amount of change in sympathetic nervous activity. In this clinical test, they used 200-mg FA daily for 2 weeks in 16 healthy subjects [186]. Matsuyama et al. [185] conducted a clinical trial of FA in Alzheimer's disease. A total of 17 patients diagnosed with mild cognitive decline were administered a daily dosage of 100 M FA for 48 weeks. However, they could not find any significant variations in A*β* accumulation and cerebral degeneration or cognitive performance. Furthermore, it failed to reduce the worsening of brain atrophy or the decrease of cognitive abilities [185] (Table 5). From these clinical studies, this review suggested that more clinical trials need to be carried out to investigate the anticancer effect of FA in several cancer treatments.

## 7. Extraction, Recovery, and Storage Methods for FA

FA in plant materials exists in three forms: soluble-free, soluble-conjugated, and insoluble-bound as a component of arabinoxylan and lignocellulosic complexes [187]. To improve the effectiveness and selectivity, sophisticated methods are gradually supplanting conventional Soxhlet extraction techniques. These methods typically feature higher levels of automation and are speedier. FA has been isolated from plant sources, especially from wheat-based foods, using a variety of techniques based on accelerated solvent extraction (ASE) [187], microwave-assisted extraction (MAE) [188], ultrasound-assisted extraction (USE) [189], subcritical water extraction (SWE), and pressurized liquid extraction (PLE) [190]. FA in its free and esterified forms could be readily extracted with pressured boiling water or aqueous ethanol solution [191]. FA must be stored properly to preserve its stability and effectiveness. It should be kept out of direct sunshine and heat in a cool, dry location [192]. In general, the optimal storage temperature is lower than 25°C (77°F). Because FA is similarly sensitive to light and pH, solutions should be kept in amber-colored containers and at a neutral pH level to reduce light-induced breakdown [22].

## 8. Conclusion and Future Direction

Cancer remains to be considered one of the most formidable diseases globally, resulting in a substantial number of mortalities annually. Chemotherapeutic techniques are often regarded as highly promising and effective therapy modalities in the treatment of cancer, but it has different adverse effects on the body. Natural products and their derivatives are promising sources of anticancer lead substances due to their fewer side effect and efficacy. FA is a naturally occurring compound that acts as a chemopreventive and chemotherapeutic substance in treating and managing different types of cancer. This study investigated that FA showed remarkable anticancer effects against breast, cervical, colorectal, colon, pancreatic, lung, liver, osteosarcoma, prostate, hepatocellular, esophageal, and glioblastoma cancer. FA showed anticancer effects against different cancer cell lines via numerous mechanisms, including oxidative stress, cytotoxicity, apoptotic cell death, cell cycle arrest, antiproliferative, autophagy, genotoxic and mutagenic, and anti-invasion pathways. However, according to the study, it is also indicated that FA exhibits its anticancer properties via regulating numerous signaling pathways, including PI3K/AKT, p38/MEK/ERK, AMPK-mTOR, P-STAT6, NF-*κ*B, and GSK signaling pathways. Although there is no clinical evidence of FA in cancer diagnosis, FA is a nontoxic substance, and limited doses have failed to show any harmful side effects or toxicity in preclinical and clinical tests in different types of diseases. However, FA showed low oral bioavailability, which is affected by the liver's fast conjugation process. This limitation can be overcome by utilizing nanotechnology, namely, through the nanoformulation of FA. In conclusion, further comprehensive clinical investigations are recommended to establish the compound as a prospective drug for the management of cancer.

## Figures and Tables

**Table 1:** Botanical sources of ferulic acid.

**Plant name**	**Plant part(s)**	**Reference**
*Arachis hypogaea* L.	Vegetables	[45]
*Avena sativa* L.	Whole grains	[46]
*Bambusa vulgaris*	Shoots	[47]
*Beta vulgaris*	Roots	[48]
*Brassica oleracea*	Vegetables	[49]
*Citrus paradisi*	Fruits	[50, 51]
*Citrus sinensis*	Fruits	[41]
*Daucus carota* subsp. sativus	Vegetables	[52]
*Hordeum vulgare* L.	Extracts	[53]
*Malus domestica*	Peel and pulps	[37]
*Musa acuminata*	Roots	[54]
*Oenanthe crocata*	Vegetables	[55]
*Oryza sativa* L.	Roots	[56]
*Persea americana*	Fruits	[57]
*Phaseolus vulgaris* L.	Beans	[58]
*Prunus domestica* L.	Fruits	[59]
*Rheum rhabarbarum* L.	Fruits	[60]
*Secale cereale* L.	Whole grains	[61, 62]
*Solanum lycopersicum* L.	Fruits	[63]
*Triticum aestivum* L.	Whole grain	[64]
*Vaccinium arctostaphylos* L.	Fruits	[65]
*Zea mays* L.	Whole grains	[66]

**Table 2:** Anticancer activity of ferulic acid.

**Type of cancer**	**Experimental model/cell line**	**Tested concentrations**	**Efficacy, IC**_**50**_ ** (exposure time)**	**Anticancer effects and mechanisms**	**Reference**
Breast cancer	4T1 cell line, MTT assay, in vitro	500 *μ*g/mL	—	↓Cell proliferation ↑Cytotoxicity	[115]
	MDA-MB-231 cell, in vitro	3–100 *μ*M	—	↑Cytotoxicity, apoptosis ↓Cell proliferation, cell migration, metastasis, tumor volume and weight, EMT process, cell growth	[116]
	MDA-MB-231 xenograft mouse model in female BALB/c nude mice, in vivo (*n* = 6)	100 mg/kg (p.o.)			
	MCF-7, MDA-MB-231, and HS578T cell lines, in vitro	25–75 *Μ*m of feruloylhexyl-amide (HFA)	—	↑Cytotoxicity, cell death ↓Cell proliferation	[117]

Cervical cancer	Hela and CaSki cervical carcinoma cell lines, in vitro	4 *μ*M	2 *μ*M	↑Cell cycle arrest in G0/G1 phase, p53 and p21 ↓Cell invasion, MMP-9, mRNA expression, cyclin D1 and cyclin E levels, LC3-II, Beclin-1, Atg12–Atg5, autophagy, proliferation	[129]
	HeLa and CaSki cells, MTT assay, in vitro	4–20 *μ*M	—	↑Cytotoxicity, apoptosis, pro-caspase-3, pro-caspase-8, and pro-caspase-9, PARP, Bax, ROS, DNA condensation ↓Bcl-2 and Mcl-1, AKT, PI3K phosphorylation	[99]

Colorectal cancer	Human CRC cell line HCT 15, in vitro	100–250 *μ*g/mL	154 *μ*g/mL	↑Cytotoxicity ↓EGFR gene expression, cell proliferation	[118]

Colon Cancer	CT-26 cell, in vitro	800 *μ*M	800 *μ*M	↑Autophagy, apoptosis, ERK, JNK, cytotoxicity, Bax ↓Proliferation, cell growth, tumor size and weight, Bcl-2, NF-*Κ*B, TNF-*α*, IL-1*β*	[119]
	BALB/c mice, in vivo (*n* = 8)	40 and 80 mg/kg (i.p.)			
	Adult male Wistar albino rats, in vivo (*n* = 10)	50 mg/kg	—	↑p53, apoptosis, caspase-3, Ki67, CK20 ↓Proliferation	[133]
	Caco-2 cell, in vitro	150 *μ*M	—	↑RABGAP1, CEP2, SMC1L1 ↓Cell proliferation, cell cycle progression	[156]
	Caco-2 cell, in vitro	1500 *μ*M	—	↑Length of the S phase, cell cycle arrest at S phase ↓Cell proliferation	[128]
	HCT116, Caco-2, and HT29 cells, in vitro	100–400 Nm dose of tributyltin (IV) ferulate	—	↑Cytotoxicity, G2/M cell cycle arrest, cell death, autophagy, LC3-II, p62 ↓RIPK1	[120]

Pancreatic cancer	MIA PaCa-2 cell, in vitro	150–750 and 1 *μ*M	500 *Μ*m/mL at 72 h	↑Cytotoxicity, p53, Bax, caspase-3, and caspase-9 ↓Colony formation, cell invasion, migration, Bcl-2, CCND1, CDK 4/6, caspase-8, and caspase-10	[121]

Lung cancer	A549 cell, in vitro	50–1000 *μ*M	—	↓Superoxide production, cell adhesion, proliferation, migration	[106]
Colon cancer	HT29-D4 cell, in vitro				

Osteosarcoma cancer	143B and MG63 cells, in vitro	10–150 *μ*M	59.88 in 143B and 66.47 *μ*M in MG63 at 48 h	↑Apoptosis, Bax, caspase-3 ↓CDK 2, 4, and 6, proliferation, Bcl-2	[134]
	MG63 xenograft model in mice (s.c.) with MG63 cells (2 × 10^6^), in vivo	100 mg/kg (p.o.)			
	SaOS-2 and MG63 cell lines, in vitro	40 *μ*M	—	↑Apoptosis, caspase-3 protein, Bax ↓Procaspase-3, Bcl-2	[135]

Prostate cancer	PC-3 and LNCaP cells, in vitro	20 *μ*M	300 and 500 *μ*M	↑Apoptosis, ATR, ATM, CDKN1A, CDKN1B, E2F4, RB1, and TP53 ↓Cell proliferation, gene expressions of CCND1, CCND2, CCND3, CDK 2, 4, and 6, colony formation, invasion	[32]

Breast cancer, liver cancer	MCF-7 and HepG2 cell lines, MTT assay, in vitro	100–200 *μ*g/mL	75.4 and 81.38 *μ*g/mL	↑Apoptosis, cytotoxicity, caspase-8 and caspase-9, annexin V levels	[122]

Hepatocellular carcinoma	HepG2 cells, in vitro	100 *μ*g/mL	—	↑Autophagy, apoptosis, caspase-3, Beclin-1, LC3-I/LC3-II, PINK-1, Parkin ↓Cell proliferation, cell growth, MMP	[136]
	Male Wistar rats, in vivo (*n* = 5)	25 and 50 mg/kg	—	↑p53, Nrf2 ↓Serum *α*-FP, AKT/PKB, NF-*Κ*B, TNF-*α*, GST, SOD, CAT, MDA, GSH, ALT, AST	[107]

Esophageal cancer	TE-4 and EC-1 cells, in vitro	20−60 *μ*M	40.98 and 40.73 *μ*M	↑Cell death, ROS, cytotoxicity, LDH release, caspase-3, apoptosis, ↓Invasion, SOD, cell growth, colony formation, migration, angiogenesis	[108]

Lung cancer	A549 cell, in vitro	—	—	↑p53 ↓Proliferation, invasion, migration, IL-4, PDGF, ↓GM-CSF, P-JAK2, ↓P-STAT6, surviving, XIAP, metastasis, COX-2	[94]
	H1299 cell, cell viability, and proliferation assay, in vitro	0.06–0.6 *μ*M of *trans*-ferulic acid (TFA)	—	↑ROS, hydrogen peroxide, superoxide anion, apoptotic, cell death, Bax ↓Cell growth, proliferation, migration, MMP2 and MMP9, surviving, colony formation, AIG capacity	[109]
	A549 cells, in vitro	0.2–50 *μ*M FXS-3	50 *μ*M	↑Apoptosis, cell cycle arrest at the G0/G1 phase, cytotoxicity, JNK ↓Cell proliferation, migration and invasion, Bcl-2, MMP2 and MMP9, ERK/p38, AKT/mTOR and MEK/ERK pathways, pulmonary tumor metastasis	[123]
	Xenograft mouse model in male BALB/C nude mice (A549 cells) in vivo (*n* = 8)	25–100 mg/kg FXS-3			

Glioblastoma	LN229, T98G, and U87 cells, in vitro	1–250 *μ*M	37.9, 34.4, and 35.1 *μ*M	↑Cytotoxicity	[124]

Breast cancer, lung cancer, cervical cancer	MCF-7, MDA-MB-231, A549, HepG2, and HeLa cells, in vitro	—	07.49 *μ*M 07.28 *μ*M 07.11 *μ*M 08.32 and 07.14-*μ*M dose of ferulic acid amides	↑Cytotoxicity	[125]

*Note:* Arrows (↑ and ↓) show an increase and decrease in the obtained variables, respectively. MMP9: matrix metalloproteinase-9; Atg5: autophagy protein 5; caspase: cysteine-aspartic acid protease; PARP: poly-ADP ribose polymerase; Bax: Bcl-2-associated X; Bcl-2: B cell lymphoma protein 2; Mcl-1: myeloid cell leukemia 1; AKT: protein kinase B; PI3K: phosphatidylinositol-3 kinase; JNK: c-Jun NH2-terminal kinase; NF-*κ*B: nuclear factor kappa-light-chain-enhancer of activated B cells; IL-1*β*: interleukin-1*β*; p53: tumor protein P53; Ki67: antigen Kiel 67; CK20: cytokeratin 20; RABGAP1: RAB GTPase activating protein 1; RIPK1: receptor-interacting serine/threonine-protein kinase; CCND1: cyclin D1; ATR: ataxia-telangiectasia; Nrf2: nuclear factor erythroid 2–related factor 2; JAK2: Janus kinase 2; STAT6: signal transducer and activator of transcription 6.

Abbreviations: CDK, cyclin-dependent kinase; EGFR, epidermal growth factor receptor; ERK, extracellular signal–related kinase; GST, glutathione S-transferases; PDGF, platelet-derived growth factor; ROS, reactive oxygen species; SOD, superoxide dismutase; TNF-*α*: tumor necrosis factor alpha; XIAP, X-linked inhibitor of apoptosis protein.

**Table 3:** Anticancer activity of ferulic acid with other molecules in combination therapy.

**Name of the compound**	**Type of cancer**	**Experimental model/cell line**	**Tested concentrations**	**Efficacy, IC**_**50**_ ** (exposure time)**	**Anticancer effects and mechanisms**	**Reference**
The combination of 2-deoxy-D-glucose (2DG) and FA	Lung cancer	NCI-H460 non–small cell lung carcinoma cells, in vitro	FA (53.8 *μ*M) and 2DG (4 *μ*M)	—	↑p21, p53, Bax, GADD45A, cytotoxicity, caspase-3, cell death, sub-G_0_ phase cells ↓DNA repair, NF-*κ*B	[166]
FA combined with epirubicin	Breast cancer	MDA-MB-231, in vitro	FA (10 and 100 *μ*M) and epirubicin (0.02–4 *μ*M)	—	↑Apoptosis, Bax, caspase-3, ER stress-related protein expression ↓Bcl-2, proliferation	[165]
FA and gamma radiation	Cervical cancer	HeLa and ME-180, MTT assay, in vitro	1–40 *μ*g/mL	10 *μ*g/mL	↑Radiation effects, TBARS, CD and LHP, levels of lipid peroxidation, ROS levels, oxidative DNA damage, cytotoxicity ↓Number of colonies, survival	[170]
FA combined with aspirin	Pancreatic cancer	MIA PaCa-2 and Panc-1, in vitro	5–5000 *μ*M	—	↑Apoptotic cell death, p-RB, p21, and p-ERK1/2, cytotoxicity ↓PCNA and MKI67, growth of tumor	[169]
		Pancreatic tumor xenograft mouse model, in vivo	FA and ASP (75 and 25 mg/kg, p.o.)			
FA combined with thyoquinine	Breast cancer	MDA-MB-231 cells, in vitro	TQ (50 and 100 *μ*M) and FA (450 *μ*M)	—	↑Apoptosis ↓Proliferation, S phase, G2/M phases	[92]
FA combined with some phenolic and flavonoids	Breast cancer, colon cancer	MCF-7 and Caco-2 cell lines, in vitro	—	19.47 and 14.20 *μ*g/mL, respectively	↑Cytotoxicity, cell death, Bax, caspase-9, p53 ↓Proliferation, Bcl-2	[168]
FA combined with P-coumaric acid	Colorectal cancer	HT-29, HCT116, and SW620 cells, in vitro	60, 120, and 180 *μ*g/mL	67.00 *μ*g/mL	↓PKM2, cell growth, aerobic glycolysis, ATP production	[171]
		Male C57BL/6J mice, in vivo (*n* = 5)	10−60 mg/kg (i.g.)			
FA combined with 4-vinylguaiacol	Colorectal cancer	HCT-116 and HT-29 cell lines, in vitro	1.5 *μ*M	350 *μ*M	↑Apoptosis, caspase-3, caspase-8, and caspase-9, cell death ↓G1phase, proliferation, cell growth, S phase, G2/M phases	[167]
FA combined with caffeic and coumaric	Hepatocellular carcinoma	HepG2 cells, in vitro	25−800 *μ*g/mL	869.16 ± 14.21, 782 ± 91.79, and 736.66 ± 13.61* μ*g/mL	↑Cytotoxicity, apoptosis ↓hTERT gene	[164]
Com with ligustrazine and tetrahydropalmatine	Endometrial cancer	Female C3H mice, in vivo (*n* = 6)	90−360 mg/kg (i.m.)	839.30 ± 121.11 or 483.53 ± 156.91* μ*g/mL, 625.20 ± 59.52 or 250.30 ± 68.12* μ*g/mL	↓MMP2 and MMP9, invasion, metastasis, ectopic volume, cell growth ↑TIMP-1	[172]
		hEM15A and HEC1-B cells, in vitro	2500 *μ*g/mL			
Ferulic acid (FA) and IR combination treatment	Lung cancer, liver cancer	A549 and HepG2 cells, in vitro	10–400 *μ*M	180 and 200 *μ*M, respectively	↑ROS level, Keap1 level, cytotoxicity, mitochondrial apoptosis, p53, p21, G2/M phase arrest, cyclin B1, mitotic arrest ↓Proliferation, p-AKT/pp38MAPK, NF-jB/p65, Nrf2, MMP9, VEGF, STAT3, cell cycle progression, Cox-2, Cdc25C	[173]
Ferulic acid combined with gemcitabine	Prostate cancer	PC-3 cell lines, in vitro	—	200 *μ*M	↑Apoptosis ↓Metastasis	[174]

*Note:* Arrows (↑ and ↓) show an increase and decrease in the obtained variables, respectively. Bax: Bcl-2-associated X; GADD45A: growth arrest and DNA damage inducible alpha; PKM2: pyruvate kinase muscle isozyme M2; Cox-2: cyclooxygenase-2; Cdc25C: dual-specificity phosphatase cell division cycle-25C.

Abbreviations: MAPK, mitogen-activated protein kinase; ROS, reactive oxygen species; TBARS, thiobarbituric acid reactive substance.

**Table 4:** Anticancer activity of different ferulic-loaded nanoformulations.

**Name of the compound**	**Type of cancer**	**Experimental model/cell line**	**Tested concentrations**	**Efficacy, IC**_**50**_ ** (exposure time)**	**Anticancer effects and mechanisms**	**Reference**
FA-NS (1:4)	Breast cancer	MCF-7 and 4T1, in vitro	5–1000 *μ*M	250 *μ*M	↑Apoptosis, lipid peroxidation, cytotoxicity, cell death ↓Cell proliferation	[178]

Ferulic acid*–*encapsulated electrospun PLGA/PEO nanofibers	Breast cancer SA	MCF-7 and HEK-293 cells, MTT assay, in vitro	1–8 wt%	—	↑Apoptosis ↓Proliferative activities	[179]

FA-loaded CS-TPP NPs	Cervical cancer	ME-180 cell, in vitro	5–80 *μ*M	40 *μ*M	↑Apoptotic cell death, cytotoxicity, cytocompatibility	[86]

PTX (8.3%)–loaded PFA nanoparticles	Colon cancer	CT26 cells, in vitro	200 *μ*L	0.79 *μ*g/mL	↑Apoptosis, ↓proliferation, tumor growth	[180]
		Xenograft (s.c. with CT26 cells) colon tumor–bearing mouse model in male BALB/c mice, in vivo (*n* = 5)	PTX, 5 mg/kg			

ZnONPs with ferulic acid (FA)	Liver cancer	Huh-7 cell, in vitro	FA (0.05–50 *μ*g/mL) and ZnONPs (10–150 *μ*g/mL)	8.2 *μ*g/mL	↑ROS, DNA damage, autophagy, apoptosis cell cycle arrest in S-phase, cytotoxicity ↓ALT, AST, ALP, *γ*-GT, TBARS	[122]
		HepG2 cell, in vitro		7.2 *μ*g/mL		

Combination of Dox/FA-PLGA-TFA NPs	Breast cancer	Female Sprague–Dawley rats, in vivo (*n* = 8)	40 and 80 mg/kg (s.c.)	—	↓P-gp level, Notch1, Hes1, Wnt-3a, *β*-catenin, MMP9, cyclin D1, ER*α*, PR, HER2, serum ALT	[87]

Chitosan-coated trans-resveratrol (RSV) and ferulic acid (FER)–loaded SLNs	HT-29 cells, in vitro	100 *μ*g/mL			↑Apoptosis, G0/G1 phase, Bax, p53 ↓Cyclin D1 and E, CDK 2, 4, and 6, MMP, Bcl-2	[181]

Ferulic acid lipid NCs	Colorectal cancer	HCT-116 and Caco2 cells, in vitro	—	—	↑Apoptosis, Bax, antiangiogenic, antioxidant, and anti-inflammatory ↓Bcl-2, MDA, IGF II, VEGF, cyclin D1	[182]
		Rats, in vivo				

*Note:* Arrows (↑ and ↓) show an increase and decrease in the obtained variables, respectively. HER2: human epidermal growth factor receptor 2; IGF II: insulin-like growth factor II; *γ*-GT: gamma-glutamyl transferase.

Abbreviations: ALT, alanine transaminase; ALP, alkaline phosphatase; AST, aspartate aminotransferase; ER*α*, estrogen receptor *α*; MMP, matrix metalloproteinase; PR, progesterone receptor; TBARS, thiobarbituric acid reactive substance; VEGF, vascular endothelial growth factor.

**Table 5:** Data of clinical trials in various diseases.

**Disease**	**Gender**	**Dose/duration**	**Mechanism/result**	**References**
Hyperlipidemia	Forty-eight hyperlipidemic subjects aged 20–60 years	1000 mg daily	↑ HDL-C ↓BAP, cholesterol, LDL-C, triglyceride, oxidative stress biomarker, MDA, LDL-C, inflammatory marker hs-CRP, TNF-*α*	[184]
Alzheimer's disease	In a study of 17 mild cognitive decline patients, 10 were assigned to the treatment group and 7 to a control group	100 M every day for 48 weeks	There are no significant variations in A*β* deposition, brain shrinkage, or mental abilities. It did not slow brain loss or impairment of cognition	[185]
Skin barrier function (SBF)	16 healthy subjects were categorized into two groups (*n* = 8)	200 mg daily for 2 weeks	↑Stratum corneum hydration strengthens SBF in healthy men ↓*Trans*-epidermal water loss, the amount of change in sympathetic nervous activity	[186]

*Note:* Arrows (↑ and ↓) show an increase and decrease in the obtained variables, respectively. MDA: malondialdehyde.

Abbreviations: BAP, biological antioxidant potential; HDL-C, high-density lipoprotein cholesterol; hs-CRP, high-sensitivity C-reactive protein; LDL-C, low-density lipoprotein cholesterol; TNF-*α*, tumor necrosis factor alpha.

## Data Availability

No new data were generated.
